# First Language Lexical Attrition in a First Language Setting: A Multi-Measure Approach Testing Teachers of English

**DOI:** 10.1007/s10936-024-10068-7

**Published:** 2024-03-06

**Authors:** Yueqingzhou Ma, Norbert Vanek

**Affiliations:** 1 University of Auckland, Auckland, New Zealand; 2 School of Cultures, Languages and Linguistics, University of Auckland, Bldg. 207, 18 Symonds Street, Auckland, 1010 New Zealand

**Keywords:** First language attrition, Chinese teachers of English, Word recognition, Lexical comprehension, Language production, Video description, Lexical diversity, Density, Sophistication, Accuracy

## Abstract

Research on first language (L1) attrition typically focuses on immigrant populations in their second language (L2) environment, yet we know comparably little about L1 attrition in the L1 setting. This study used two lexical tasks to test L1 attrition, a time-sensitive word decision task and a video retelling. Chinese teachers of English vs. Chinese teachers of other subjects (*N* = 25/group) were recruited at a secondary school in China. The aim was to provide an exploratory basis of the L2 influence on L1 lexical attrition in the L1 environment, both on the level of lexical comprehension and production. Mixed-effects models were used to analyse multiple measures including response accuracy and reaction times in comprehension, and lexical diversity, density, sophistication, and accuracy in production. The results showed Chinese teachers’ L1 lexical attrition in the form of longer response times to high-frequency Chinese words compared to non-English Chinese teachers, and the use of significantly fewer sophisticated words in their retellings. Also, teachers of English were faster and more accurate in decisions about Chinese borrowings from English, suggesting L2-driven influence on their mental lexicon. Considering participants’ background information, analyses showed that increased L2 exposure and frequency of use can predict L1 lexical attrition.

## Introduction

Language attrition can be viewed as a reversal process of language acquisition resulting from reduced or no language contact (Schmid & De Bot, 2004). This process is a non-pathological and non-permanent loss of a language in a bilingual individual (Sorace, 2020), exhibited in receding access to some grammatical or lexical features. Classifications vary based on which language is lost, distinguishing between L1 attrition and L2 attrition, or also based on the environment where a language is lost, namely loss of L1 in an L1 environment, loss of L1 in an L2 environment, loss of L2 in an L1 environment, and loss of L2 in an L2 environment (Van Els, 2012). L1 attrition has received much attention in academia, but rarely in the L1 environment. One explanation of the imbalance (e.g., Seliger &Vago, 1991) may stem from the assumption that L2 users who live in an L1-dominant environment enjoy rich L1 exposure with little risk of L1 attrition. However, Schmid and Köpke (2009) reasoned that in bilinguals acquiring or using an L2, the L2 may interfere with and change how the L1 system is accessed. In other words, it may seem that the L1 environment can keep the L2 users’ L1 system relatively stable, with signals of L1 attrition present but less noticeable than in an L2 environment.

Language attrition can affect different components of a linguistic system (morphology, phonology, syntax, semantics) in a different order (first/last), at a different speed (slower/faster), and to different extents (less/more) (Paradis, 2007). This phenomenon may also be sensitive to external factors such as the age of acquisition, length of residence, and L1 or L2 exposure (Schmid & Jarvis, 2014). An intuitively convincing assumption is that the lexicon is the largest system of linguistic knowledge, and it is an open-class domain where networks of lexemes are less densely connected and interdependent than other linguistic components such as the phonological inventory and grammatical features (Schmid & Köpke, 2009; Schmid & Jarvis, 2014). Schmid and Jarvis (2014) investigated L1 lexical attrition in two groups of German L1 attriters with Dutch or English as L2. The study compared controlled, elicited and free speech data with more established measures (type/token frequencies) and well as more sensitive distributional measures of, among others, the relative rarity of words used. The results of lexical analyses showed that less controlled elicitation could differentiate attriters and non-attriters better, and that weakening in L1 lexical diversity was too subtle to be detected just via type/token ratios. One implication is that if L1 attrition in the L1 environment is even less perceptible than in an L2 context, then the lexicon in less controlled tasks provides an ideal testbed as it is the most vulnerable part of the linguistic system and is the first, fastest, and most dramatically deteriorating component (Montrul, 2008; Schmid & Jarvis, 2014). Further implications from extant research on L1 attrition include the necessity to consider multiple aspects of attrition and incorporate real-time processing (Sorace, 2020). To the best of the authors’ knowledge, processing studies focusing on the topic of Mandarin language attrition in China are currently absent.

To address this research gap, this study offers a psycholinguistic analysis of comprehension and production performance with the aim to investigate L1 (Mandarin) lexical attrition in Chinese teachers of English based in China. This choice was based on the current linguistic context in China, where English is the dominant L2, with over 200 million users and with 50 million secondary school students studying it in 2016 (Zhang, 2016). This context provides Chinese ESL teachers with both more opportunities and pressure to keep improving their English language skills. Such a pressure goes with seeking extended immersion in English, which is in turn likely to interact with L1 Mandarin and bring about the possibility of L1 attrition. Here we explore two layers of possible L1 lexical attrition of Chinese teachers of English, L1 attrition in comprehension and production, as well as the role of individual factors affecting L1 attrition. Our target group consists of 25 Chinese teachers of English (the ENT group) and our control group are Chinese non-English teachers (the NENT group). The study was driven by the rationale to investigate whether Chinese ESL teachers with long-term exposure to English would exhibit L1 lexical attrition in their comprehension, tested via a word decision task, and production, tested via a video retelling.

To generate suggestions for theoretical advancement in the field of L1 attrition, individual difference factors, including age, level of education, and amount of language exposure were considered, following the Multicomponential view (Köpke et al., 2007). Out of these components, level of education may be the most critical factor influencing L1 attrition (Jaspaert & Kroon, 1989), with the rationale that a higher education level may provide language users more solid knowledge of linguistic structures, which would be less vulnerable to attrition. We also consider the view that language attrition can be affected by the typological distance between the bilinguals’ two languages (Park, 2018). The logic is that L1 attrition may be more likely to occur when L1 and L2 are similar (Altenberg, 1991) rather than with huge typological distance between the L1 and L2 (Ecke, 2004) where there are fewer shared linguistic structures and thus less interference (Köpke et al., 2007). Interference and its links to L1 attrition can be explained via the Competition Model (MacWhinney, 1997), which views increased L2 input as a trigger that fine-tunes the activation weights governing language processing. Increased L2 exposure means decreased L1 activation. Language competition and fine-tuning of activation weights in the bilingual mind is also compatible with De Bot’s Dynamic System Theory (DST) (De Bot, 2007). Under the DST, L1 and L2 dynamically interact and L1 lexical attrition may result from such interaction. Another important link to theory is the Critical threshold hypothesis (Neisser, 1984), postulating a distinct line between language acquisition and language attrition. Ecke (2004) advocated that a language is not easily attritted when it becomes highly proficient. Once a critical threshold has been reached, the linguistic system stabilises, and it may become largely but not fully immune to interference or decay. L1 attrition may still occur, but with a more stabilised linguistic system it may be less likely. For increased validity, the extent of decay is best measured via multiple measures.

## Background to the Study

### Measuring Lexical Attrition

Lexical attrition involves two layers of analysis, lexical comprehension and production. Various methods are used to test lexical comprehension, including word recognition, picture-word matching, and a lexical decision task (LDT). Word recognition is a process in which participants need to identify words and word parts. Its relevance to attrition is that if bilinguals correctly recognise a smaller number of words or need more recognition time than monolingual controls, relative slowdowns and accuracy decreases can serve as indicators of lexical attrition. In production, methods vary from unscripted speech, verbal fluency tasks (VFT) to picture naming. Unscripted speech enables participants to talk about a selected topic, and transcriptions of verbal output can be used to analyse the distribution and frequencies of words and word types in one’s productive lexicon. The rationale for using unscripted speech is not only through quantity but also through the quality or *lexical richness*, measuring the variety and complexity of vocabulary in language samples. Lexical richness is a multidimensional construct defined in multiple ways. Arnaud (1984) defined lexical richness as a composite of three categories, lexical diversity, lexical sophistication, and lexical errors. Read (2000) further refined the definition of lexical richness to comprise lexical variation, lexical density, lexical sophistication, and lexical accuracy. The present study combines earlier approaches and defines lexical richness as four constructs, including lexical diversity, density, sophistication, and accuracy.

*Lexical diversity* is the variety of active vocabulary deployed by a speaker or writer. It is a suitable measure to reflect the extent of lexical knowledge, following the rationale that speakers or writers who are more proficient, and have more lexical items at their disposal, are able to use a great variety of words, while less proficient speakers or writers often tend to repeat a smaller number of lexical items (Read, 2000). Lexical diversity is indexed by type-token ratio (TTR), calculated as the total number of different words in a text (types) divided by the total number of words in a text (tokens). This measure was criticised because it was shown to be strongly correlated with text length, wherein the longer text samples, the less lexical diversity in that text (e.g., McCarthy & Jarvis, 2007). Covington and McFall (2010) proposed a more objective algorithm labelled MATTR (Moving Average Type-Token Ratio). MATTR is a more robust approach which eliminates the effects of text length by calculating TTR through a pre-set length of a moving window (Hřebíček et al., 2014). TTR is used in this study as an indicator of lexical diversity, and to avoid text-length effects we employed MATTR (Covington & McFall, 2010).


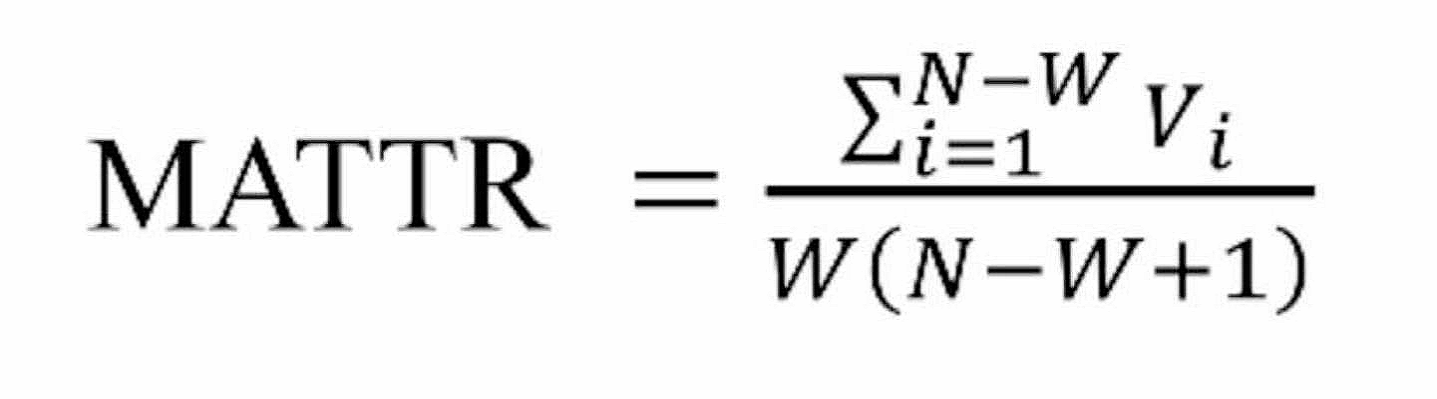


*Lexical density* refers to the ratio that relates the number of content words to the number of total words in a text (Ure, 1971). As criteria for distinguishing function words and content words, one might define function words in English to include articles, pronouns, prepositions and conjunctions, and content words to include nouns, verbs, adjectives and adverbs (Halliday & Christie, 1989). Following Huang and Liao (2007), Modern Chinese uses a slightly different taxonomy. Compared with English, the types of content words in Chinese form a richer category (nouns, verbs, adjectives, attributives, numerals, classifiers, adverbs, onomatopoeias, interjections) while function words form a narrower category (prepositions, conjunctions, particles, modal particles). This distinction is adopted for the present study to calculate lexical density ratios in texts.


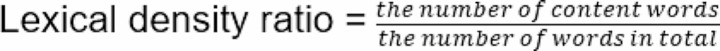


*Lexical sophistication* captures both the depth and breadth of lexical knowledge available to speakers and writers (Meara, 1996). This term also refers to the number of low-frequency words suitable for the topic and the style of writing (Read, 2000). Lower frequency of occurrence of a word in a natural language implies higher degrees of sophistication. One common method to establish word frequencies is to look at representative corpora (e.g., McNamara et al., 2009). In this approach, a reference corpus (e.g., the British National Corpus) is chosen first, and then it is used as a reference to calculate the frequency of each word that occurs in a target text. The average frequency score for a text is created by dividing the sum of all frequency scores by the number of words in the target text (Kyle & Crossley, 2014). This approach can also be used to predict the degree of lexical sophistication. However, this approach is not sensitive to how widely a particular word is used. Thus, Range (also known as dispersion) is proposed to measure the breadth of word use, calculated by providing a total of the number of documents in which that word occurs. One way to assess lexical sophistication in English texts is through *Range*. Range is a software (Heatley et al., 1994, 2002) that includes the Most Frequent 1000 Word List, the Second Most Frequent 1000 Word List, and Academic Word List. At this point, Range has no version for assessing lexical sophistication in Chinese texts. Following the logic in Range, this study used a word list from HSK (Hanyu Shuiping Kaoshi, Chinese Proficiency Test, 2001) vocabulary and Chinese character grade outline (hereafter Outline, 2001) to assess lexical sophistication in Chinese texts (Wu, 2016).

*Lexical accuracy* denotes the error rate when using the lexicon. Lexical errors can be form-oriented or content-oriented (Hemchua & Schmitt, 2006), which is a distinction using a grammatical/linguistic criterion. The definition of lexical errors under the grammatical/linguistic criterion refers to a deviation in form and/or meaning of the word in the target language. Form deviations not only include orthographic or phonological deviations within the boundaries of a single word but also violations of syntactic constraints. Meaning deviations include semantic inconsistencies, i.e., situations when a lexical item is attributed a new meaning in a particular context, and this attribution disobeys the word’s semantic restrictions (Llach, 2011). Considering the unique features of Modern Chinese, Zhang (2007) classified lexical errors in written Chinese into form errors (e.g., wrong characters) and meaning errors (e.g., polysemes unfit for the given context). The present study adopts Zhang’s criterion to measure lexical errors in Mandarin.

## Methodology

This study used two tasks, a word decision task to test lexical comprehension and a video retelling to test production. Performance in the two tasks was compared between Chinese teachers of English (the target group) with that of non-English teachers (the control group) with the aim to test the manifestations of L1 lexical attrition in Chinese teachers of English. The research question was: To what extent is the L1 lexicon of Chinese teachers of English attrited?

### Participants

The target group included 25 Chinese teachers of English (the ENT group) with over five years of teaching experience in a secondary school in China, and the control group involved 25 Chinese non-English teachers (the NENT group) in the same school. The school where the participants were recruited offers English as a foreign language, not as the medium of instruction. The ENT group was at the time of testing in daily contact with English, preparing their students for the College Entrance Examination. The NENT group self-reported that they had had very limited contact with spoken or written English in daily life between the time of their graduation and this study. The ENT group included 22 females and 3 males, with an average age of 40.28 years (range 27–54), with the average length of teaching of 17.8 years (range 5–36), two participants with an MA degree, and the rest with a BA degree. The NENT group included 10 females and 15 males, with an average age of 42.24 years (range 23–59), with the average length of teaching of 17.8 years (range 1–40), two participants with an MA degree, twenty with a BA degree, and two without a degree.

In terms of Chinese language knowledge, all ENT participants were PSC-certified (a national test for Mandarin known as ‘Putonghua Shuiping Ceshi’, which is an oral test to examine teachers’ standard degree and proficiency using Mandarin, divided into six levels from 1-A as the highest level to 3-B as the lowest level). In the ENT group, thirteen participants got 2-A, and twelve participants got 2-B, while in the NENT group nine participants got 2-A and sixteen participants got 2-B. In both groups, participants began to formally learn Mandarin at an early age (ENT: sixteen participants started with Mandarin in the kindergarten, eight in 1st or 2nd grade at the primary school, and one participant in secondary school; NENT: ten participants started with Mandarin in the kindergarten, twelve in 1st or 2nd grade at primary school; and three participants in secondary school). Most participants had passed the L1 College Entrance Examination (scored out of 150) (ENT: eleven participants with 90–110 points, seven participants with 110–129 points, three participants with 130–150 points; NENT: twelve participants with 90–110 points, six participants with 110–129 points, four participants with 130–150 points). As for Mandarin use in daily life, all participants used their L1 with high frequency (ENT: fourteen participants were exposed to Mandarin for over 80 h/week and seven reported 50-to-80 h/week; NENT: twenty reported being exposed to Mandarin over 80 h/week, three reported 50-to-80 h/week).

As for English language knowledge, most ENT participants were certified upper-intermediate/advanced users (six with CET4-6 and12 with TEM 4–8, while CET stands for College English Test administered by the Higher Education Department of the Ministry of Education in China, and TEM stands for Test for English Majors, which is mandatory for students with English majors). However, most NENT participants lacked proficiency in English (twenty-one participants did not have language certification of any level, while only four passed CET4-6). In terms of age of onset, most ENT participants (*N* = 23) started learning English formally in secondary school, two at an early age (3–5 grade) in primary school. In the NENT group, sixteen participants started with English formally in secondary school, six at an early age (3–5 grade) in primary school. Most ENT participants passed the English exam as part of college entrance examination, fifteen with grades 110–129/150; four with 90–109; three with high grades of 130–150, while only three failed this exam (below 90). In comparison, most NENT participants passed the English exam in the College entrance examination, but the number of high scorers was smaller. Three participants scored 130–150, four scored 110–130 and over half scored 90–109/150 (three failed). All the ENT participants came from the same province (Sichuan), so regional differences in college entrance examinations had no effect on the comparability of participants’ L1 and L2 scores. As for frequency of use, almost all ENT participants (24) used English at work or study and ticked mostly. In the NENT group, most participants (24) ticked seldom for use of English. Regarding the amount of weekly L2 English exposure, over half of the ENT group reported increased exposure to L2 English (seventeen participants reported over 80 h/week, three reported 50-to-80 h/week, and four reported 20-to-50 h/week). In contrast, the NENT group’s exposure to English was typically under 20 h weekly (twenty-two reported less than 20 h/week). No participant in either group had English immersion experience. The rationale for choosing teachers was their substantial daily contact with English. Overall, the ENT and the NENT group differed in general English proficiency, the frequency of use of English, as well as the amount of exposure to English at the time of testing. The sum of these between-group differences was deemed sufficient for potential L1 attrition effects in the ENT group to surface.

### Materials

The materials for the comprehension task (speeded lexical decision task) consisted of 45 Chinese words and 45 Chinese nonwords. All 45 words were content words that comprised two characters. The 45 words were subdivided into 15 high-frequency Chinese words, 15 low-frequency words and 15 Chinese borrowings from English. The 15 high-frequency words and 15 low-frequency words were selected from the Frequency Table of Words in Modern Chinese Corpus (Xiao, 2010). The high-frequency words (e.g., 生产 ‘produce’, 时间 ‘time’) had an average word frequency of 350.2 per million and an average number of strokes of 15.5. The low-frequency words (e.g., 书籍 ‘book’, 覆盖 ‘cover’) had an average word frequency of 13 per million and an average number of strokes of 21.9. The Chinese borrowings from English (e.g., 克隆 ‘clone’, 蹦极 ‘bungee’) were selected from the Modern Chinese Dictionary (2012). Low-frequency words were expected to take longer to respond to because of more sophisticated strokes. All 45 Chinese nonwords comprised two characters and all conformed to the construction principles of Chinese character orthography. There were three Chinese nonword types to ensure variation in complexity, namely fifteen no-meaning nonwords, fifteen nonwords with one non-existing character, and fifteen nonwords with two non-existing characters. Fifteen no-meaning nonwords were combinations of two characters selected from the Chinese words list. The characters of these fifteen no-meaning nonwords exist in Mandarin Chinese, but the nonwords themselves are meaningless. No nonword of this type appears in the Modern Chinese Dictionary (2012). The other two types of nonwords, with one or two non-existing characters, were screened prior to the experiment to ensure that the characters were non-existent in Chinese. The rationale for including carefully controlled nonwords in the lexical decision task was threefold, namely to provide control items when testing the extent to which between-group performance in the speed of lexical access might differ, to see how easily the distinction between lexical vs. non-lexical information gets recognised in the two groups, and to isolate the likely effect of factors like word length and nonword status (existing/non-existing characters) when tracking the efficiency and accuracy of lexical recognition processes in possible L1 attriters. The full lists of words and nonwords are available on the project website https://osf.io/zc7mv/.

The material for the production task (video retelling) was a three-minute cartoon without verbal input, called The Flying Cat (retrieved from https://www.youtube.com/watch?v=GOOGFAd8820). The video in this task was paused every 30 s to give the participants about two minutes to write down what happened in the video they had just seen. The lexical decision task as well as the video retelling task were piloted before the main experiment. For verification purposes, three participants from each group completed the tasks, and their results showed that the instructions were clear, and the instruments worked as planned. No adjustments were needed, so the data from the piloting stage were included in the analyses.

### Procedure

Participants had three tasks to complete, a language background questionnaire, a word decision task and a written story description. For the word decision task (using Psychopy 3.0), participants were tested individually, and they were first asked to read the instructions on a computer screen. The instructions were to look at Chinese lexical items appearing one at a time on a screen and decide whether it was or it was not a Chinese word. They were asked to press the “←” key on the keyboard if they thought what they saw was a Chinese word or the “→” key if they thought it was not a Chinese word. They were instructed to decide as quickly and accurately as possible. The experiment started only after the participants confirmed that they had understood the task and procedures. This task took approximately 5 min to complete. The logic of the timed word decision task was to measure the speed and accuracy of decisions about (non)words, assuming that lexical access difficulties emerging as slower and less correct decisions could signal L1 lexical attrition. Combining an *online* measure of reaction times with an *offline* measure of accuracy scores allowed us to tap into the moment-by-moment manifestations and the outcome once processing has occurred, respectively (Sato & Vanek, 2023). Combinations of online and offline measures in a single study have fruitfully informed research in domains other than language attrition as well, including the processing of negation (Zhang & Vanek, 2021) or emotional expressions (Vanek & Tovalovich, 2022).

For the story description task, participants watched the chosen nonverbal cartoon on an electronic whiteboard in a classroom. All participants completed the task in a single session. They were asked to write down in Chinese what happened in the video shown to them. The video was paused every 30 s to minimise potential effects of differences in memory capacity and to ensure the written descriptions could be as detailed as possible. This task took approximately 20 min to complete. Participants were given an opportunity to view and comment on their descriptions at the end of the task, but they could not change their descriptions. All procedural steps and design features of this study were approved by the University of Auckland Human Participants Ethics Committee, Ref. UAHPEC22630.

## Results

We first present the results from the word decision task results using two measures - accuracy and reaction times, followed by the results from the video retelling using four measures - lexical diversity, density, sophistication, and accuracy. All results are outputs of analyses of linear mixed effects regression models with group as the main predictor.

### Accuracy of Lexical Decisions

Accuracy in the word decision task is first compared as the percentage of correct responses averaged per group and lexical item type (words vs. nonwords). When a Chinese word was shown on the screen, a left arrow keypress counted as an accurate response, and when a Chinese nonword was shown, a right arrow keypress counted as an accurate response. Accuracy in nonword trials means that a participant correctly identified a given nonword item as not a Chinese word. On average, Chinese nonwords were responded to less accurately in the ENT group (*M* = 67.1, *SD* = 22.20) than the NENT Group (*M* = 78.4, *SD* = 12.3). However, no differences emerged when the two groups made decisions about existing Chinese words (*M* = 96.3, *SD* = 22.30 for ENT; *M* = 96.2, *SD* = 12.36 for NENT). In the next step, we used R (Version 4.1.0; R Development Core Team, 2018) and lme4 package (Baayen et al., 2008) to verify through a linear mixed-effects analysis whether the observed between-group difference is statistically significant. We entered Lexical type and Group and their interaction as fixed effects factors, and Participant and Lexical item as random effects factors. To check the contribution of Lexical type, we also built a reduced model excluding Lexical type and compared it with the full model. The model comparison showed that the inclusion of Lexical type significantly increased the model fit (*χ2* (1) = 9.34, *p* < 0.05), confirming that response accuracy for words was significantly higher than for nonwords. To check the contribution of Group, we also built a reduced model excluding Group and compared it with the full model. The model comparison showed that the presence of Group significantly increased the model fit (*χ2*(1) = 4.40, *p* < 0.05), confirming that response accuracy for NENTs was significantly higher than for ENTs. Crucially, the interaction between Group and Lexical type in the full model was significant too (*β* = -11.4, *SE* = 5.27, *t* = -2.165, *p* < 0.05), showing that it was the nonwords in particular that were driving the accuracy differences between the two groups.

We next examined accuracy scores for individual word and nonword types in more detail. The three types of Chinese words were (a) borrowings from English, (b) high-frequency Chinese words, (c) low-frequency Chinese words. Accuracy scores were similar across groups for high-frequency words (*M* = 98.4, *SD* = 5.29 for ENT; *M* = 98.4, *SD* = 10.54 for NENT) and low-frequency words (*M* = 98.0 for, *SD* = 5.24 for ENT; *M* = 97.2, *SD* = 10.54 for NENT). However, the ENT group was more accurate in decisions about borrowings from English (*M* = 93.6, *SD* = 5.41) than the NENT group (*M* = 87.8, *SD* = 10.52). Then, a linear mixed-effects analysis of the relationship between Words and Group was conducted to test the effects of different language groups (ENT vs. NENT) on the accuracy in deciding about specific types of Chinese words. Words and Group were specified as fixed factors, Participant and Lexical item as random factors (accuracy ∼ words + group+ (1| participant) + (1| lexical item)). To look at the effect of word type, we compared the full model with the reduced model excluding word type. The comparison showed that the inclusion of word type significantly increased the model fit (*χ2*(2) = 11.11, *p* < 0.05), with borrowings driving the difference. In the next step, the reduced model excluding Group compared to the full model showed that the presence of Group also significantly increased the model fit (*χ2*(2) = 4.22, *p* < 0.05), with ENT group being more accurate. Importantly, the interaction between Group and Word type in the full model was significant too (*β* = 9.0, *SE* = 2.65, *t* = 3.4, *p* < 0.05), and pairwise comparisons confirmed that it was the higher accuracy of the ENT group than the NENT group for the English borrowings that was driving the interaction.

**Fig. 1:**
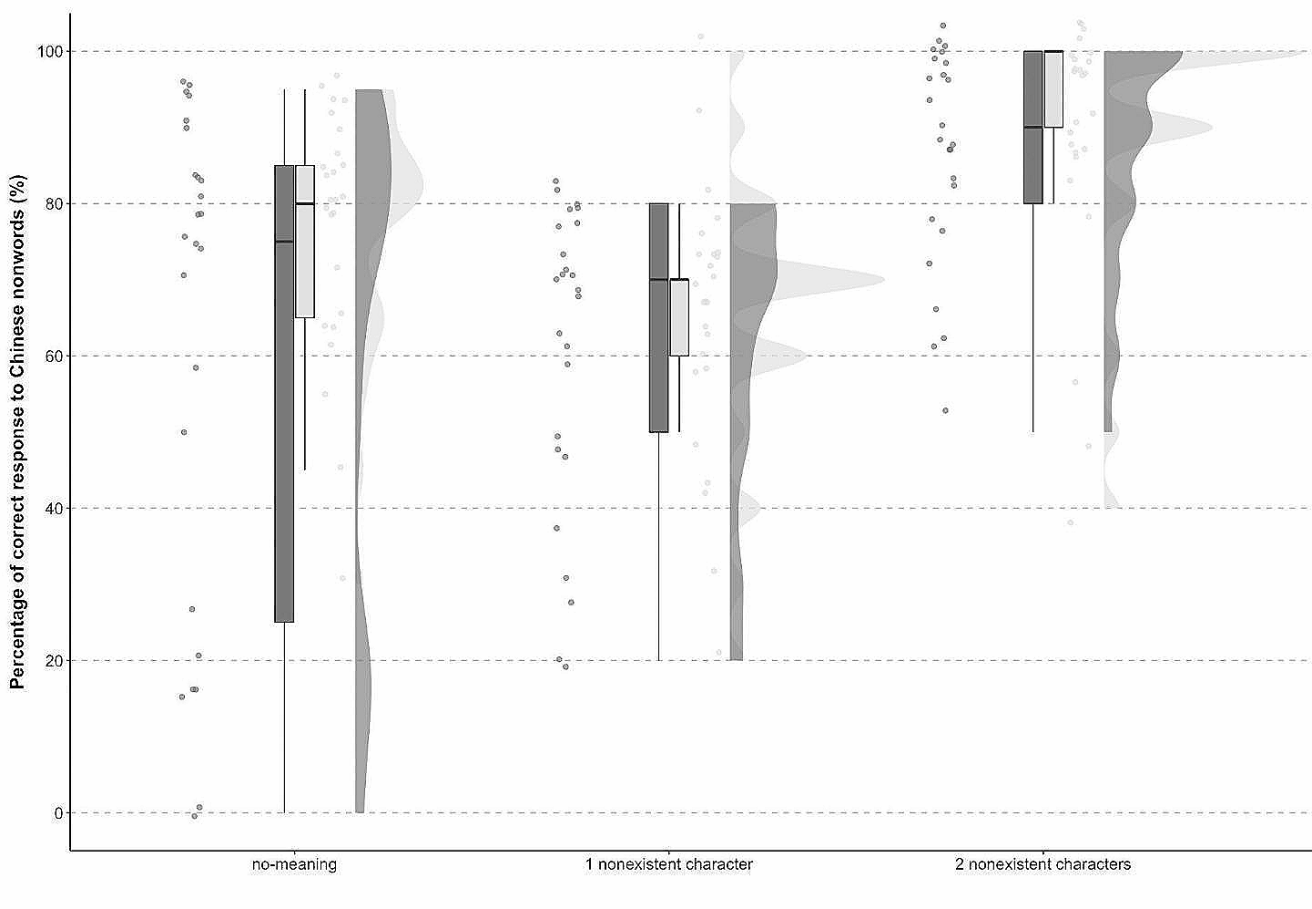
Accuracy scores for English (ENT) and non-English teachers (NENT) in lexical decisions about three types of nonwords (no-meaning, one non-existent character, and two non-existent characters). Box plots show the medians and the 50% of the correct lexical decision times within the boxes. Raincloud and violin plots were added to aid visualisation of the data distribution patterns across conditions and groups

Accuracy differed even more robustly for Chinese nonwords (the proportions of correct answers are shown in Fig. 1). There were three different types of Chinese nonwords, i.e., no-meaning, one non-existent character, two non-existent characters. Overall, responses were most accurate for nonwords with two non-existent characters, followed by no-meaning and one non-existent character nonwords respectively. For nonwords with two non-existent characters, the ENT group were less accurate (*M* = 86.8, *SD* = 25.95) than the NENT group (*M* = 89.6, *SD* = 17.85). For no-meaning nonwords, the ENT group was also less accurate (*M* = 62.0, *SD* = 26.62) than the NENT group (*M* = 76.8, *SD* = 19.71). And for nonwords with one non-existent character, the ENT group was less accurate (*M* = 60.8, *SD* = 25.83) than the NENT group (*M* = 64.4, *SD* = 18.93).

A linear mixed-effects analysis with Nonwords and Group as fixed factors was built and compared with reduced models, following the logic described earlier. A reduced model excluding Nonwords compared with the full model showed that the presence of Nonwords significantly increased the model fit (*χ2*(2) = 12.11, *p* < 0.05), with correct rejections for nonwords with one non-existent character being the lowest and with two non-existent characters being the highest. However, unlike Nonwords, the inclusion of Group in the model did not significantly contribute to the model fit. (*χ2* (1) = 1.96, *p* > 0.05), showing that accuracy rates within individual nonword types were not group-specific.

### Response Times

To add a more sensitive indicator of lexical access, response times (RT) were compared across groups and conditions. Overall, the ENT group took longer to correctly respond to Chinese nonwords (*M* = 1465.30, *SD* = 636.38) and words (*M* = 1450.55, *SD* = 645.94) than the NENT group (*M* = 1393.04, *SD* = 510.58 for nonwords; *M* = 1380.23, *SD* = 510.58 for words). In the next step, mixed effect models with the same logic as earlier were built to check the statistical significance of these between-group differences. A reduced model without Lexical type compared with the full model returned a significant difference (*χ2*(1) = 8.302, *p* < 0.05), confirming that, overall, participants reacted to nonwords more slowly than to words. Then, we also compared a reduced model excluding Group with the full model with. This comparison did not yield a statistically significant result (*χ2*(1) = 0.552, *p* > 0.05), suggesting that RTs of the participants per condition did not vary based on group.

**Fig. 2:**
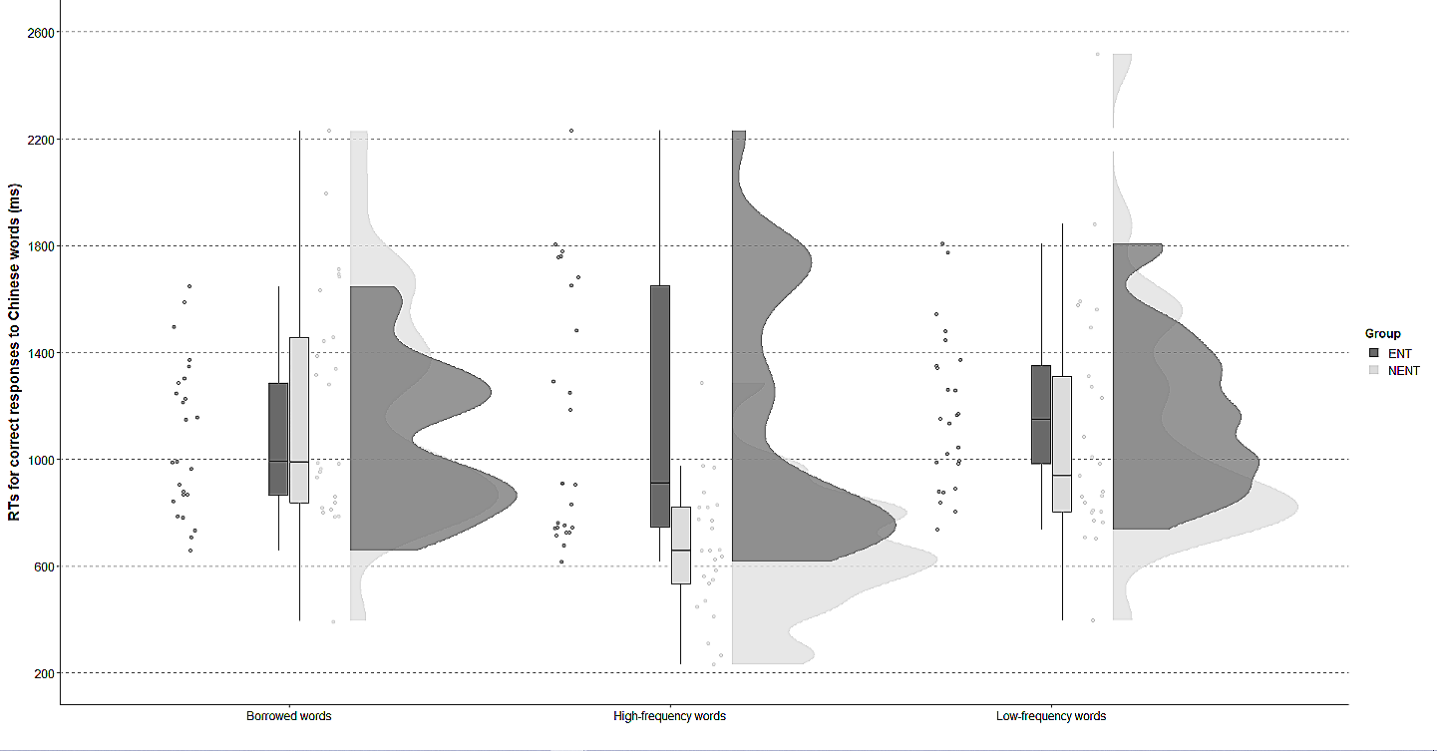
Response times for English (ENT) and non-English teachers (NENT) in lexical decisions about three types of words (borrowings, high-frequency, low-frequency)

Looking into the three different types of Chinese words (borrowings, high-frequency, low-frequency) more closely, we observed a marked between-group difference in the category of high-frequency words (Fig. 2). The ENT group needed longer to correctly decide about high-frequency words (*M* = 1145.15, *SD* = 357.95) than the NENT Group (*M* = 658.88, *SD* = 457.93). The between-group difference was less pronounced for low-frequency words, with ENTs still somewhat slower (*M* = 1172.63, *SD* = 290.14) than NENTs (*M* = 1105.98,0.14, *SD* = 461.28). The pattern was reversed for borrowings from English, with the ENT group (*M* = 1081.51, *SD* = 284.4) outperforming the NENT group (*M* = 1203.30, *SD* = 446.71).

Next, we built a mixed-effect model with the fixed effects of Group and Words and their interaction (RTs ∼ words*group+(1|participant)+(1|lexical item)) to test how RTs for different types of Chinese words vary. The interaction between Group and Words in the full model was significant (*β* = -609.06, *SE* = 142.56, *t* = -4.27, *p* < 0.05), suggesting that response speed in some word types was group-specific. To explore the nature of the interaction, we next built a reduced model excluding Words. The model comparison showed that the presence of Words was a significant contributor to the variation in the RT data (*χ2*(1) = 7.24, *p* < 0.05). Then, a reduced model excluding Group compared with the full model returned a significant result too (*χ2*((1) = 4.29, *p* < 0.05), showing that Group membership was also a significant factor affecting RT patterns. Pairwise comparisons looking at individual word types showed that the ENT group responded significantly slower for high-frequency words (*F*(1,48) = 20.43, *p* < 0.05) than the NENT group. In other word types, between-group differences were not significant, either for the low-frequency words (*F*(1,48) = 0.37, *p* > 0.05) nor for borrowings (*F*(1,48) = 1.34, *p* > 0.05). We also compared the RTs for correct decisions about different types of Chinese nonwords. On average, between-group differences were negligible and did not reach significance (*χ2*(1) = 0.02, *p* > 0.05), indicating that response speed did not vary based on group membership.

### Lexical Diversity, Density, Sophistication, and Accuracy

Lexical diversity was calculated by means of the type-token-ratio (TTR). The number of tokens in the descriptions ranged from 100 to 600. Text length varied; thus we applied the Moving Average Type-Token Ratio (MATTR) formula to control for potential text length effects on the TTR. Each text was segmented using the automatic part-of-speech tagging system (Xiao, 2016). We then checked the segmentation and tagging results manually, following the classification of modern Chinese words (Huang & Liao, 2007). The segmented and tagged texts were fed into MATTR software (Covington & McFall, 2010) to get the MATTR score of every piece of text. The window size was set to 100 to consider minimal token numbers. On average, the ENT Group produced texts with slightly lower lexical diversity (*M* = 0.47, *SD* = 0.03) than the NENT group (*M* = 0.48, *SD* = 0.04), but this difference was not statistically significant (*F*(1,48) = 2.44, *p* > 0.05).

**Fig. 3:**
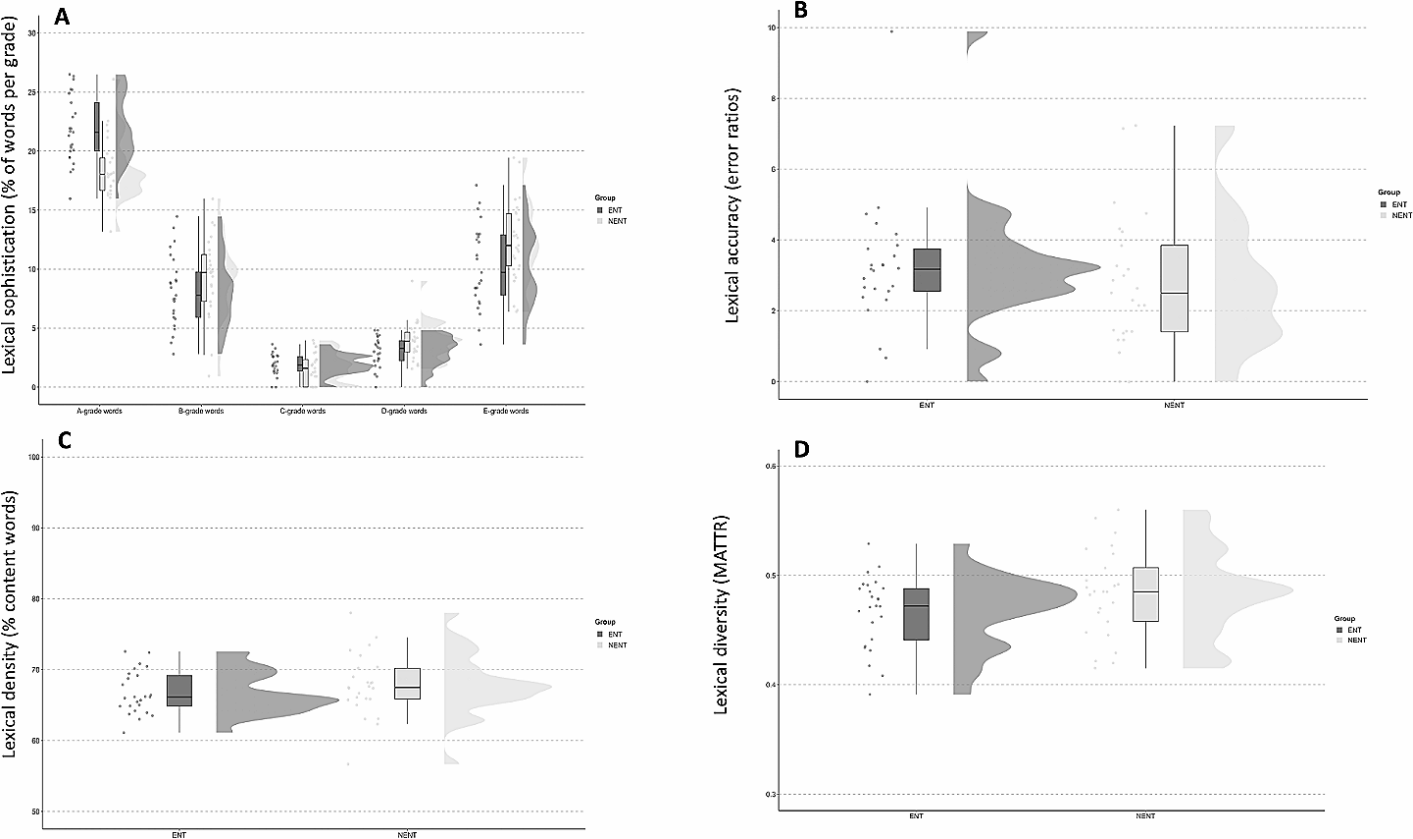
(A) Lexical sophistication shown as the mean ratio of five word types and the total number of words per group. (B) Lexical accuracy shown as the mean percentage of errors from the total number of words in texts. (C) Lexical density shown as the mean ratio of content words and the total number of words per group. (D) Lexical diversity illustrated as the average MATTR score per group

Lexical density expresses the ratio of content words and the number of total words in a text (Ure, 1971). Following Huang and Liao (2007), words in Chinese were counted as content words if they were expressed by nouns, verbs, adjectives, attributive words, numerals, classifiers, adverbs, pronouns, onomatopoeias, and interjections. To assess the level of lexical density, we calculated the number of total words and content words using AntConc (Anthony, 2021). Then we calculated the proportion of content words in each text, and the averages per group and participant are shown in the raincloud plots in Fig. 3. On average, the lexicon in the descriptions written by the ENT Lexical density was very similar across groups (ENT: *M* = 66.71, *SD* = 3.03; NENT: *M* = 67.79, *SD* = 4.38). A linear model of content word percentages changing as a function of group showed that this difference was not statistically significant (*F*(1,48) = 1.04, *p* > 0.05).

Lexical sophistication was assessed using word lists available in Outline (2001). The word list involved four grades of Chinese words, i.e., 1033 A-grade words, 2018 B-grade words, 2202 C-grade words, and 3569 D-grade words, with A-grade words as the least and D-grade words as the most sophisticated. To avoid potential effects of text length, we operationalised lexical sophistication as the proportion of word tokens from A-grade, B-grade, C-grade, D-grade, and the word tokens not included in the Outline (E-grade) to the total number of tokens in a text. The proportions per group are shown in Fig. 3.A. On average, the ENT group used more A-grade (least sophisticated) words (*M* = 21.79, *SD* = 2.83) than the NENT group (*M* = 18.46, *SD* = 2.66). The proportions of B and C grade words were comparable across the two groups. D-grade words were less frequent in the ENT group (*M* = 3.03, *SD* = 1.28) than in the NENT group (*M* = 3.86, *SD* = 1.64). Regarding E-grade words (i.e., the most sophisticated grade), the ENT group used fewer (*M* = 10.27, *SD* = 3.52) than the NENT group (*M* = 12.33, *SD* = 3.44). Linear models of word percentages changing as a function of group confirmed statistically significant between-group differences for A-grade words (*F*(1,48) = 18.45, *p* < 0.05), D-grade and E-grade words (*F*(1,48) = 5.69, *p* < 0.05; *F*(1,48) = 4.28, *p* < 0.05), but not for B-grade and C-grade words (*F*(1,48) = 1.56, *p* > 0.05; *F*(1,48) = 1.08, *p* > 0.05).

Lexical accuracy expresses the percentage of lexical errors from the total word count in a text. Both form and meaning errors were included and so were miswritten characters, self-corrections, and deletions. On average, the lexical error ratio of the ENT group (*M* = 3.22, *SD* = 1.82) was higher than that of the NENT group (*M* = 2.78, *SD* = 1.90), suggesting that the ENT group performed worse in terms of lexical accuracy than the NENT group, but this difference entered in the linear model was not statistically significant (*F*(1,48) = 0.70, *p* > 0.05).

## Discussion and Conclusions

### Manifestations of L1 Lexical Attrition in Lexical Decisions and Production

Two manifestations of L1 attrition emerged in the two tasks. In the lexical decision task, when correctly deciding about high-frequency Chinese words, the ENT group needed significantly more time to react than the NENT group. In the video retelling, there were significant differences in lexical sophistication between the two groups, with a marked decrease in sophisticated expressions in the ENT group.

Delays in decisions about high-frequency words in the ENT group are interpreted as a sign of lexical attrition. High-frequency word attrition describes a situation when the words that bilinguals had previously known are difficult to retrieve (Jarvis, 2019). Retrieval can eventually be successful, but the extraction of these words from memory may undergo an effortful and relatively time-consuming mental search. Greater difficulty retrieving and accessing the mental lexicon can qualify as an L1 attrition effect (Schmid & Jarvis, 2014) resulting from an increased cognitive load for the ENT group who need to manage two linguistic systems simultaneously (Schmid & Köpke, 2009). It was particularly the high frequency Chinese words where automaticity of access exhibited delays. Not only did bilinguals need more time to retrieve high-frequency L1 lexical items, but their inaccuracies were also more pronounced, particularly when deciding about nonwords. This may be due to an increased number of lexical competitors for bilinguals when deciding if something is a word or not. In sum, the ENT group deciding more slowly about high-frequency L1 words and less accurately about nonwords than the NENT are two findings interpreted as manifestations of L1 lexical attrition in comprehension.

In production, we consider L1 lexical attrition phenomena by examining lexical richness in four different dimensions, lexical diversity, density, sophistication, and accuracy. The findings point to lower lexical diversity, density, and accuracy in the ENT group compared to the NENT group, but these differences were not statistically significant. These findings do not fully align with Liu and Lin’s findings (2013). Liu and Lin (2013) also used a video retelling task to compare L1 lexical richness of two groups of China-based college students (i.e., English major versus non-English major), and observed significant differences in lexical accuracy between the two groups. That is, the English-major group had lower lexical accuracy than the non-English group. This difference in results may be attributable to participants’ L2 exposure type. While Chinese teachers of English in this study teach intermediate English in secondary schools, college English-major students may be exposed to more advanced discussions in L2 and specialised English language knowledge, which may increase interference from English on L1 lexical accuracy. Unlike for accuracy, major between-group differences were observed in lexical sophistication. The ENT group tended to use a higher proportion of less sophisticated words and a smaller proportion of highly sophisticated words than the NENT group. This reduced sophistication could imply a lower breadth of lexical knowledge available in their written descriptions (Meara, 1996, 2005). It may stem from internal changes of the L1 system, reflecting that the L1 system is simplified or attrited to a measurable degree.

### On the Less Noticeable Manifestations of L1 Lexical Attrition

One aspect of lexical decisions where the ENT outperformed the NENT group was in faster and more accurate decisions about lexical borrowings from L2 English. This advantage can be attributed to increased contact with and better knowledge of English. When L1 lexical attrition is viewed sequentially, L2 influence on the L1 is likely to precede. The finding that the ENT group could decide about borrowings faster and with greater accuracy than the NENT group implies that the mental lexicon of the ENT group may have begun to diverge from the L1 pattern under the influence of the L2, albeit not necessarily entirely (Pavlenko, 2011). Divergence from the L1 was also noticeable in video descriptions, when more ENTS than NENTs resorted to alphabetic text. This signals that the English frame of reference, even though less noticeably, was more active in the ENT than in the NENT group. Also, a speed advantage in response to borrowings does not mean that the ENT group’s L1 is less accessible. Borrowings from L2 are not viewed as a threat to the L1, particularly when they are integrated into the phonological and orthographic system, such as soda 苏打, and become a part of the L1 (Liu & Lin, 2013). Perhaps this is also why lexical diversity of the ENT group did not exhibit significant changes. Highlighting the ENT group’s advantage in processing borrowings is important to balance often negativistic approaches to examine attrition in bilinguals in terms of what their linguistic systems lack rather than what they already have (Schmid, 2011). Changes in the L1 system due to language contact are multi-layered, which is why the approach promoted here is to examine the subtlety of changes simultaneously in both directions, both in what is lost and what is gained.

A multi-measure approach is in a good position to shed light also in the less noticeable manifestations of attrition including lexical density, diversity and accuracy. Although the related between-group differences were not statistically significant, on average, the ENT group was less accurate in decisions about the three types of Chinese nonwords than the NENT group. Furthermore, Fig. 1 also shows that compared to the relatively consistent NENT group, variation in the ENT group’s decision about Chinese nonwords was considerably greater. This increased variation might be linked to different degrees of interference between the ENT’s L1 and L2, which implies there may be differences in the levels of L1 attrition.

### Factors Influencing First Language Lexical Attrition

We adopt the Multicomponential view (Köpke et al., 2007) to explain first language attrition, including the major factors (age, educational level, typological differences between languages, the frequency of language use, and language proficiency). First, regarding age of L1 acquisition, the background information questionnaires showed that all participants from the ENT group started to learn Mandarin Chinese before puberty (16 in kindergarten, 8 in 1st-2nd grade at primary school; and 1 in secondary school), suggesting their L1 linguistic system was relatively stable and not easily attritable. Another reason for less noticeable manifestations of attrition is the observation that bilinguals in the L1 environment exhibit first signs of L1 attrition at the age of around 40, noticeable phenomena at around 50, and the most noticeable L1 attrition at around 70 (Goral, 2004). However, in this study the mean age of the ENT group was 40.28 years (range 27 to 40, with only one participant aged 54). If at this age the L1 linguistic system is not typically interfered with by their L2, it could explain why the ENT group exhibited no noticeable manifestations of attrition in their L1 lexical density, diversity, and accuracy.

Second, Jaspaert and Kroon (1989) argued that the level of education is the most important explanatory factor of L1 attrition. The participant base in this study was comparable across groups in terms of education level across groups, all with postgraduate degrees in the ENT group (2 MA, 23 BA) and most in the NENT group (1 MA, 23 BA and only 1 sub-BA). An overall high educational level in the L1 is indicative of high L1 literacy across groups, which can explain the absence of significant between-group differences in lexical density, diversity, and accuracy. Nevertheless, the ENT group’s frequent use and exposure to L2 may have resulted in crosslinguistic interference, exhibited as increased reaction times in decisions about high-frequency L1 words and decreased frequency of sophisticated L1 word use. Future research may benefit from adding educational level as a fixed factor in models to see whether it can reliably predict the degree of L1 attrition.

Third, L1 attrition is less likely to occur in bilinguals whose L1 and L2 are typologically distant (Ecke, 2004; Köpke et al., 2007). In the present study, the L1 (Mandarin) and L2 (English) boast numerous typological differences, with very few lexical items sharing form across Chinese and English. Assuming that the L1 and L2 compete when bilinguals retrieve words from memory, not much competition occurs when L1-L2 lexical forms are very different. Over time, little need to compete requires relatively low levels of L1 inhibition, keeping the L1 system active in the bilingual’s mind. The present findings align with these ideas, with the ENT group demonstrating an ability to correctly decide about Chinese words accurately and without significant disadvantages in lexical density and diversity. To move the agenda forward, future research could contrast typologically close and distant L1-L2 pairs within a single study to establish the relationship between typological distance and attrition effects more firmly. Future work could find it beneficial to eliminate the limitations of the present study, for instance by recruiting participants with a lower degree of variation within groups, and participants with fewer similarities shared between groups (in terms of age/educational level/ L1-L2 typology/L2 frequency/L2 proficiency).

### Theoretical Implications for L1 Lexical Attrition in an L1 Context

The amount of L1 use is often intuitively regarded as the most important factor in determining L1 attrition (Köpke et al., 2007), with less exposure to L1 leading to more L1 attrition over time (Köpke, 1999). In this study, over half of the ENT group were exposed to the L1 more than 80 h weekly, which qualifies as rich L1 contact comparable to that of the NENT group. In other words, L1 exposure of the ENT group cannot be the driving factor of the observed between-group differences since the ENT group’s L1 exposure was not decreased. However, what differs between groups is the increased amount of L2 exposure in the ENT group. Over half of the ENT group reported increased exposure to L2 English while the NENT group’s exposure to English was typically under 20 h/week. Faster and more accurate reactions to Chinese borrowings from English in the ENT group may be attributable to crosslinguistic influence of the L2 on the L1 (Park, 2018). The set of findings that point to L1 lexical attrition in the ENT group, including decreased reaction time for high-frequency L1 words, and lower proportion of sophisticated word use, can also be assigned to L2 influence, more specifically to L2 interference. Under the interference hypothesis, L1 attrition is caused by the newly dominant or competing language. When languages compete in the bilingual mind (MacWhinney, 1997), input in more than one language can trigger fine-tuning of the activation weights governing language processing. As the bilinguals’ L2 exposure increases, L1 activation decreases and more noticeable manifestations of L1 attrition may occur.

Under the Dynamic System Theory (De Bot, 2007), the growth or decline of a specific language system depends on the resources and interaction between input and internal forces. For the ENT group, their L1 exposure remains considerable while their L2 exposure has increased. Increased L2 input may interfere with the L1, leading to L1 attrition in some aspects of language processing, while the undiminished amount of L1 exposure can simultaneously be buffering attrition effects in other areas of L1 processing. Such dynamicity between the L1 and L2 is in a good position to explain the mixture of noticeable manifestations of L1 attrition in some aspects of processing, (e.g., slower retrieval time for high-frequency L1 words) co-existing alongside less noticeable manifestations of L1 attrition (e.g., subtle decreases in lexical density, diversity, and accuracy).

Returning to the Critical threshold hypothesis (Neisser, 1984), language users above the critical threshold have their L1 system stabilised and largely immune to interference or decay, while in contrast, language systems below the threshold suffer from fast, severe, and extensive attrition. The questionnaire data show that the L1 proficiency of the ENT group was comparable to the NENT group. Together with the College Entrance Examination tests, the scores imply that both the ENT group and the NENT group can use standard Mandarin fluently. Another perspective on the findings of this study is through the Activation Threshold Hypothesis (ATH, Paradis, 1993), which foregrounds the role of frequency of language use as a critical factor affecting L1 attrition. The underlying premise is that access of lexical items stored in memory requires neuronal excitation. The level of energy necessary to retrieve any given item is determined by the frequency and recency of its previous retrieval. Long-term absence of activation triggers language attrition (Paradis, 2007). Non-use of the L1 lexicon means that its activation threshold will rise. Based on the frequency of L1 use alone, the predictions of the ATH do not align with the findings showing that the ENT group has decreased decision speed for high-frequency words in comprehension, and lag behind the NENT group’s use of sophisticated words in production. For a more satisfactory account, one also needs to consider the impact of increased L2 use on the activation threshold of the L1. Increases in the activation threshold of some of the L1 (high-frequency and more sophisticated lexicon) may be attributed to a substantial increase in L2 use. Alternating L2 and L1 use raises the L1 activation threshold in the ENT group due to more frequent inhibition of the L1 when the L2 is used. Reactivation of the inhibited L1 incurs cognitive costs in the form of longer reaction times and reduced sophistication.

In sum, given the complex nature of language attrition, a range of factors needed to be considered in explaining related phenomena, including linguistic and extralinguistic factors (Park, 2018). Our selection of linguistic factors and their link to manifestations of L1 lexical attrition is not exhaustive, we zoomed in on the amount of L1 and L2 exposure, L1 proficiency, frequency of L1 and L2 use, and typological differences between the two languages. As for extralinguistic factors, we filtered out the ones with testable predictions for L1 attrition from the literature, namely age and educational level. Our findings challenge the intuitively appealing idea that L1 attrition in the L1 environment only occurs when the speakers are advanced L2 learners or do not use their L1 for a long time, thereby leading to no or few noticeable manifestations of L1 attrition in the L1 environment (Seliger & Vago, 1991). On the contrary, our findings show that combining production and comprehension tasks with a multi-measure approach can help to uncover a range of manifestations of first language attrition in a first language environment.

## References

[CR1] Altenberg, E. P. (1991). Assessing first language vulnerability to. In H. W. Seliger, & R. M. Vago (Eds.), *First language attrition* (pp. 174–188). CUP.

[CR2] Anthony, L. (2021). *AntConc (Version 4.0.2) [Computer Software]*. Waseda University.

[CR3] Arnaud, P. J. (1984). *The Lexical Richness of L2 Written Productions and the Validity of Vocabulary Tests* Papers from the International Symposium on Language Testing (7th, Colchester, England).

[CR4] Baayen R, Davidson D, Bates D (2008). Mixed-effects modeling with crossed random effects for subjects and items. Journal of Memory and Language.

[CR5] Covington MA, McFall JD (2010). Cutting the Gordian knot: The moving-average type–token ratio (MATTR). Journal of Quantitative Linguistics.

[CR6] De Bot, K. (2007). Dynamic systems theory, lifespan development and language attrition. In B. Köpke, M. S. Schmid, M. Keijzer, & S. Dostert (Eds.), *Language attrition: Theoretical perspectives* (pp. 53–68). Benjamins.

[CR8] Ecke P (2004). Language attrition and theories of forgetting: A cross-disciplinary review. International Journal of Bilingualism.

[CR9] Goral M (2004). First-language decline in healthy aging: Implications for attrition in bilingualism. Journal of Neurolinguistics.

[CR10] Halliday, M., & Christie, F. (1989). *Spoken and Written Language* (2nd ed.). Oxford University Press.

[CR11] Heatley, A., Nation, P., & Coxhead, A. (1994). *Range*. Victoria University of Wellington. https://www.lextutor.ca/range/.

[CR12] Hemchua S, Schmitt N (2006). An analysis of lexical errors in the English compositions of Thai learners. Prospect.

[CR13] Hřebíček, L., Altmann, G., Čech, R., Macutek, J., & Uhlířová, L. (2014). *Empirical approaches to text and Language Analysis: Dedicated to Ludĕk Hřebíček on the occasion of his 80th birthday*. Beltz.

[CR15] Huang, B. R., & Liao, X. D. (2007). *Xiandai Hanyu [Modern Chinese]*. Higher Education Press.

[CR16] Jarvis, S. (2019). Lexical attrition. In M. Schmid, & B. Köpke (Eds.), *The Oxford handbook of language attrition*. Oxford University Press.

[CR17] Jaspaert K, Kroon S (1989). Social determinants of language loss. Review of Applied Linguistics (ITL).

[CR18] Köpke, B. (1999). L’attrition de la première langue chez le bilingue tardif: implications pour l’étude psycholinguistique du bilinguisme. (Unpublished Ph.D. dissertation). Université de Toulouse, Toulouse, France.

[CR19] Köpke, B., Schmid, M. S., Keijzer, M., & Dostert, S. (2007). *Language Attrition: Theoretical perspectives*. Benjamins.

[CR21] Liu XL, Lin LH (2013). Zhongguo Gaoji Yingyuxuexizhe Hanyu Cihui Moshi Yanjiu [A study on the attrition of Chinese vocabulary by Chinese Advanced English learners]. Foreign Language Teaching and Research.

[CR20] Llach, A. (2011). *Lexical errors and accuracy in Foreign Language writing*. Multilingual Matters.

[CR22] MacWhinney, B. (1997). Second language acquisition and the competition model. In de A. Groot, & J. Kroll (Eds.), *Tutorials in Bilingualism: Psycholinguistic perspectives* (pp. 113–142). LEA.

[CR23] McCarthy PM, Jarvis S (2007). Vocd: A theoretical and empirical evaluation. Language Testing.

[CR24] McNamara DS, Crossley SA, McCarthy PM (2009). Linguistic features of writing quality. Written Communication.

[CR25] Meara P (2005). Lexical frequency profiles: A Monte Carlo Analysis. Applied Linguistics.

[CR26] Modern Chinese Dictionary (Vol. (2012). *Institute of language, Chinese Academy of Social Sciences* (Vol. 6). Commercial.

[CR27] Montrul, S. (2008). *Incomplete acquisition in bilingualism. Re-examining the age factor*. Benjamins.

[CR28] Neisser U (1984). Interpreting Harry Bahrick’s discovery: What confers immunity against forgetting?. Journal of Experimental Psychology: General.

[CR29] Paradis M (1993). Linguistic, psycholinguistic, and neurolinguistic aspect of ‘Interference’ in bilingual speakers: The activation threshold Hypothesis International. Journal of Psycholinguistics.

[CR30] Paradis, M. (2007). L1 attrition features predicted by a neurolinguistic theory of bilingualism. In B. Köpke, M. S. Schmid, M. Keijzer, & S. Dostert (Eds.), *Language attrition: Theoretical perspectives* (pp. 121–134). Benjamins.

[CR31] Park, E. S. (2018). Language attrition. *The TESOL encyclopedia of English language teaching*, 1–12.

[CR32] Pavlenko, A. (2011). *Thinking and speaking in two languages*. Multilingual Matters.

[CR33] R Development Core Team. (2018). *R: A language and environment for statistical computing*. R Foundation for Statistical Computing.

[CR34] Read, J. (2000). *Assessing vocabulary*. Cambridge University Press.

[CR35] Sato, S., & Vanek, N. (2023). Contrasting online and offline measures: Examples from experimental research on linguistic relativity. In S. Zufferey, & P. Gygax (Eds.), *The Routledge handbook of experimental linguistics* (pp. 217–234). Routledge.

[CR37] Schmid, M. S. (2011). *Language attrition*. Cambridge University Press.

[CR38] Schmid, M. S., & De Bot, K. (2004). Language attrition. In Davies, A., & Elder, C. (Eds.). (2008). The handbook of applied linguistics (pp 210). Hoboken, NJ, United States: John Wiley & Sons.

[CR39] Schmid MS, Jarvis S (2014). Lexical access and lexical diversity in first language attrition. Bilingualism: Language and Cognition.

[CR36] Schmid, M., & Köpke, B. (2009). L1 attrition and the Mental Lexicon. In A. Pavlenko (Ed.), *The Bilingual Mental Lexicon: Interdisciplinary approaches* (pp. 209–238). Multilingual Matters.

[CR40] Seliger, H. W., & Vago, R. M. (1991). First Language Attrition (1st Edition). Cambridge, the United States: Cambridge University Press.

[CR41] Sorace A (2020). L1 attrition in a wider perspective. Second Language Research.

[CR43] Ure, J. (1971). Lexical density and register differentiation. In G. Perren, & J. L. M. Trim (Eds.), *Applications of Linguistics* (pp. 443–452). Cambridge University Press.

[CR45] Van Els, T. (2012). An overview of European Research on Language Attrition. In B. Weltens, K. Bot, T. Van, & Els (Eds.), *Language Attrition in Progress* (pp. 3–18). De Gruyter Mouton.

[CR44] Vanek N, Tovalovich A (2022). Emotionality ratings and electrodermal responses to university-related expressions in a native and a non-native language. International Journal of Bilingual Education and Bilingualism.

[CR46] Wu JF (2016). Yingyu Muyuzhe Hanyuxiezuo Zhong De cihuifengfuxing fazhan yanjiu [A study on the development of lexical richness in Chinese writing by native English speakers]. Chinese Teaching in the World.

[CR47] Xiao, H. (2010). Frequency Table of Words in Modern Chinese Corpus. Retrieved from http://corpus.zhonghuayuwen.org/Resources.aspx.

[CR48] Xiao, H. (2016). The online Chinese word segmentation and automatic part-of-speech tagging system. Retrieved from http://www.aihanyu.org/cncorpus/CpsWParser.aspx.

[CR49] Zhang B (2007). Tongyici Fanyici Yihunxiaoci: Cong hanyu dao zhongjieyu de shijiao zhuanyi [Synonyms and antonyms: A shift from Chinese to interlanguage]. Chinese Teaching in the World.

[CR50] Zhang DL (2016). Zhongguo Yingyujiaoyu De Fazhan Yu weilai [The development and future of English education in China]. Contemporary Foreign Language Studies.

[CR51] Zhang H, Vanek N (2021). From no, she does to yes, she does: Negation processing in negative yes–no questions by Mandarin speakers of English. Applied Psycholinguistics.

